# Participation restrictions and vocational rehabilitation needs experienced by persons with a unilateral lower limb amputation in the Western Cape, South Africa

**DOI:** 10.4102/ajod.v8i0.456

**Published:** 2019-06-10

**Authors:** Tak Wing Yu, Liezel Ennion

**Affiliations:** 1 Department of Physiotherapy, University of the Western Cape, Cape Town, South Africa

**Keywords:** physical therapist, lower limb amputations, participation restriction, rehabilitation, vocational rehabilitation needs

## Abstract

**Background:**

Vocational rehabilitation (VR) aims to rehabilitate a person with an amputation back into actively participating in society. Even though lower limb amputation (LLA) surgery is commonly performed in South Africa (SA), little research has been published on the participation restrictions experienced by and vocational needs of persons with LLA in the Western Cape (WC).

**Objectives:**

The aim of this study was to determine and explore the participation restrictions and VR needs of persons with a unilateral LLA in the WC.

**Method:**

A mixed-methods approach and a sequential exploratory design were utilised to collect data from 50 persons with an LLA. Participants were conveniently sampled within the Cape Metropole region of the WC, SA. The World Health Organization Disability Assessment Schedule 2.0 (WHODAS 2.0) tool was used to collect the quantitative data, and telephonic interviews were conducted for qualitative data collection.

**Results:**

A third (28%) of participants in this study were unemployed, and only 14% (*n* = 7) of the participants owned or used a prosthesis. In addition, 50% of the participants either had a disability grant or were on pension. The participation restrictions identified were mainly related to mobility where 74% (*n* = 37) of participants had extreme difficulty with mobility in general, 92% (*n* = 46) struggled with walking distances longer than 1 km and 80% (*n* = 40) had extreme difficulty in completing household tasks quickly. The main VR needs identified in this study were the inadequate rehabilitation services that target ambulation (standing and walking) to facilitate employment.

**Conclusion:**

Persons with a unilateral LLA still experience significant difficulties in mobility 3 months post-amputation, which negatively affects their participation in society and vocational activities.

## Background

South Africa (SA) has a total population of 51.8 million people of which 25% are economically active (World Bank 2017). Within the total population, 7.5% experience some kind of disability (WHO 2017). Disability is normally associated with mental or physical impairments; these impairments hinder the capability of a person to do or complete his or her tasks. It is important to have a system that is able to identify these impairments before treatment or assistance can be given. Considering that poverty predisposes disability and disability places an increased financial demand on the person with a disability, vocational rehabilitation (VR) should therefore be a priority in SA (Hughes 2003).

Vocational rehabilitation is referred to as a process of engaging and re-engaging work with a person (Escorpizo et al. 2011). Although VR will address the problem, the underlying reasons, participation restrictions of persons with a lower limb amputation (LLA), need to be identified first. To date, there is no literature available on the participation restriction or VR needs of persons with LLA in the Western Cape (WC), SA.

Persons with a disability face many challenges when interacting with their environment. Two of the most important barriers identified by the World Health Organization, Disability and Health (WHO) are the physical and environmental barriers and challenges with accessing health services (WHO 2017). Central to these challenges are usually either (1) a lack of disability-friendly facilities or (2) a mobility impairment that was not adequately addressed through rehabilitation (WHO 2017).

Persons who have undergone an LLA will experience either a temporary or a permanent decrease in mobility as a result of physical impairments in balance and proprioception or a lack of access to prosthetic technology (Bragaru et al. 2011; Yazicioglu et al. 2007). In SA where there is already limited access to healthcare and assistive technology, mobility impairments and environmental barriers would further restrict participation in society (Ennion, Johannesson & Rhoda 2017; Ennion & Rhoda 2016). The hypothesis for this study was that persons with an LLA in the Cape Metropole would not be actively participating in vocational activities such as employment, recreational sports and hobbies and therefore require more focused VR.

## Methods

The study was conducted in the Cape Metropole district in the WC province of SA. A mixed-method approach and a sequential explanatory design were utilised (Creswell 2014). The mixed-methods approach lends itself to using both quantitative and qualitative instruments in one study to gain an in-depth understanding of the research problem (Creswell 2014). For the quantitative phase, 50 participants were conveniently sampled from a tertiary government hospital (*n* = 35), a private subacute hospital (*n* = 3) and through snowball sampling (*n* = 12). Male and female participants over the age of 18 years, and at least 3 months post-major unilateral LLA, were included in the study. No participants with neurological impairments were included in the study, as it might negatively affect their participation in society and bias the study findings. For the qualitative phase of the study, eight participants were conveniently selected from the initial sample of 23 out of the 50 participants who consented to participate in an in-depth telephonic interview.

A basic demographic survey was developed by the researcher to capture information such as age, gender, race, cause of amputation, level of amputation, use of prosthesis, as well as the level of education and household income. The World Health Organization Disability Assessment Schedule 2.0 (WHODAS 2.0) tool was used to collect data regarding participation restrictions (WHO 2001). The WHODAS 2.0 is a validated and standardised Likert scale survey. The World Health Organization Disability Assessment Schedule 2.0 has eight parts in the questionnaire focusing on different areas, namely understanding and communicating, ambulation, self-care, socialising, life activities, health-related difficulties and participation in society (WHO 2001). Permission was obtained online from the WHO to use the English WHODAS 2.0 for research purposes. The participants completed the demographic survey along with the WHODAS 2.0 in the presence of a researcher who would assist them in clarifying the questions they did not understand in the survey. Once the participants had completed the WHODAS 2.0, the data were captured in the Statistical Package for the Social Sciences (SPSS version 22) for analysis. After the completion of the quantitative phase, a semi-structured, telephonic interview guide (Appendix 1) was developed based on the results of the WHODAS 2.0 (WHO 2001). The questions were aimed at exploring the underlying reasons for the challenges with mobility and participation in society (reported in the WHODAS 2.0). It further explored the participants’ experiences with post-amputation rehabilitation and identifying any VR needs that could potentially facilitate active participation in society. Out of the 50 participants who completed the WHODAS 2.0 in the quantitative phase of the study, 23 participants initially consented to participate in the second phase of the study. These 23 participants were contacted telephonically, but 12 participants could not be reached on the telephone number that they provided. A further three participants refused to continue participating in the study. Even though the optimal sample size for conducting interviews is 10–12, only eight participants were willing to participate in the telephonic interview in the second phase of the study (Creswell 2014). During the interview audio was recorded. Once the interview was completed the interviews were transcribed verbatim by the researcher and the researchers’ assistant. The WHODAS 2.0 results provided four themes: participation restriction, self-care, life activities and mobility. Within the four predetermined themes, the qualitative data were then analysed using Creswell’s (2014) six-step process of thematic analysis. The interviews were first organised and prepared for the data analysis. After preparing, the researcher read through the interviews to get a general idea of the data collected and then started to put codes next to the different items. Themes were then identified and reviewed.

Ethics clearance was obtained from the University Research Ethics Committee, and permission to access patients was obtained from the medical manager from the respective hospitals. Permission was obtained from the Provincial Department of Health (DOH) to gain access to the patients’ database and medical records at the tertiary hospital. In addition, permission was granted by the Head of the Physiotherapy Department for the researcher to come back and investigate their departmental statistics to contact patients on their database. Potential participants’ medical folders were retrieved and reviewed against the inclusion criteria to establish their suitability for the study. The same process was followed to review statistics and identify potential participants from the weekly wound clinic where persons with an LLA would return for follow-up. Suitable participants were contacted to arrange a convenient time to complete the WHODAS 2.0 at the hospital. Data were collected over a period of 12 months. The purpose of the study was explained to each participant, and written informed consent was obtained from all participants before their participation. The participants completed the demographic survey followed by the WHODAS 2.0. Additional patients were recruited from a private subacute hospital. Permission to conduct the research was obtained from the relevant departments of the private subacute hospital. In the subacute hospital’s facility, the patients were identified by the physiotherapist in charge of the ward and written informed consent to participate was obtained. Once the physiotherapist at the private subacute hospital obtained consent, the researcher was notified. The researcher then met with the patients in the ward to collect data. Furthermore, the researcher set a time frame and place with the participants who were recruited via snowball sampling, where the participants would complete the questionnaires.

### Ethical considerations

This project obtained ethical clearance from the Senate Research Committee of the University of the Western Cape (clearance number: 15/4/33).

## Results

In this study, the majority of participants (72%; *n* = 36) were males. The mean age of participants was 57 years (standard deviation [SD] ±12 years and range is 23–77 years). When considering the participants’ racial distribution, 62% (*n* = 31) were mixed race people, 26% (*n* = 13) were white people and 8% (*n* = 4) were black people (Table 1).

**TABLE 1:** Demographic details of the participants who completed the WHODAS 2.0.

Race	Male	Female	Total
Gender	36	14	50
Black people	1	3	4
Mixed race people	23	8	31
White people	10	3	13
Other people	1	0	1
Missing data	1	0	1

WHODAS 2.0, World Health Organization Disability Assessment Schedule 2.0.

The majority of participants (52%; *n* = 26) suffered a trans-tibial amputation and only 14% (*n* = 7) had a prosthesis. Of those who had a prosthesis, only one was employed. In the total population (*n* = 50), 22% (*n* = 11) of the participants were employed at the time of data collection. Furthermore, 50% (*n* = 25) of the participants received a disability grant or were on pension. The remaining 28% (*n* =14) did not fill in this section.

In the first phase, the WHODAS 2.0 was used to collect the data. There are six sections in the WHODAS 2.0 which look at different aspects of health and disability of the participants (Table 2). In both the ‘understanding and communication’ and ‘getting along with people’ sections, only 2% (*n* = 1) experienced difficulty. In the ‘getting around section’, 60% (*n* = 30) had great difficulty. In the ‘self-care’ section, 8% (*n* = 4) struggled. Only 8% (*n* = 4) of participants had problems in the ‘life activity’ section, while 22% (*n* = 11) of the participants experienced problems in the final section of ‘participation in society’. The total scoring that led to those percentages could not be calculated because of missing data.

**TABLE 2:** World Health Organization Disability Assessment Schedule 2.0 sections results.

WHODAS 2.0 sections	No. of participants that struggled	Percentage	No. of participants that did not struggle	Percentage
D1: Communication and understanding	1	2	49	98
D2: Getting around	30	60	20	40
D3: Self-care	4	8	46	92
D4: Social life	1	2	49	98
D5: Life activities	4	8	46	92
D6: Participation in society	11	22	39	78

WHODAS 2.0, World Health Organization Disability Assessment Schedule 2.0.

From the WHODAS 2.0, participants reported having most difficulty with ‘getting around’ and ‘participating in society’. When considering the individual questions under the ‘getting around’ section, 75% (*n* = 35) of the total participants had moderate to extreme difficulty with standing for a long period of time. In addition, 92% (*n* = 46) of the participants struggled with walking long distances (± 1 km). It was also identified that 80% (*n* = 40) of participants struggled to do their housework quickly. Under the ‘participating in society’ section, 60% (*n* = 30) reported to have been emotionally affected. Moreover, 62% (*n* = 31) of the population reported that they struggled to participate in relaxing (recreational activities) without the support and assistance of family members or friends (Table 3).

**TABLE 3:** Percentage of participants that had experienced difficulty in the World Health Organization Disability Assessment Schedule 2.0 sections.

Variables	None – mild	Percentage	Moderate – extreme	Percentage
**D2: Getting around**				
Standing for a long period of time	15	30	35	70
Long distance walking	4	8	46	92
**D5: Life activity**				
Doing all the household task quickly	10	20	40	80
**D6: Participation in society**				
How emotionally have you been affected	18	36	32	64
Problem in relaxation	19	38	31	62

A chi-squared test was used to identify whether there is a significant relationship between the components of the WHODAS 2.0 questionnaire. Three tests were executed: mobility (getting around) and participation in society, age and mobility, and age and participation in society.

The first chi-squared test showed a statistically significant relationship (*p* = 0.012) between scores for the mobility (getting around) and ‘participation in society’ sections of the WHODAS 2.0. To further explore the significant relationship between mobility and participation in society, age was introduced. Two chi-squared tests were performed between age and mobility. The age and mobility chi-squared test showed no significant relationship (*p* = 0.44). In the third chi-squared test with age and participation in society, it also resulted in no significant relationship (*p* = 0.197).

In the qualitative phase, eight participants participated in a semi-structured telephonic interview. Table 4 provides an overview of the demographic details of the participants in the second phase of the study.

**TABLE 4:** Demographic details of the participants in the qualitative phase.

No.	Gender	Age	Highest level of education	Race	Income (ZAR)	Employment	Disability grant
1	Male	73	University	White people	1400	No	No
2	Male	76	High school	White people	1400	No	No
3	Male	58	High school	White people	1400	No	No
4	Male	51	Primary school	Black people	1400	No	Yes
5	Male	58	Primary school	Mixed race people	1400	No	Yes
6	Female	42	University	White people	25 000	Yes	No
7	Male	70	High school	Other people	Missing	N/A	No

From the semi-structured interviews, the underlying reasons for challenges with mobility that arose were (1) poor balance, (2) an inability to stand for longer periods of time, (3) poor cardiovascular endurance which limited their walking distance and (4) fear of falling. Because of the limited number of participants who were employed and the challenges with mobility that most participants experienced, very few VR needs could be identified from the interviews. Participants generally expressed a need for (1) prolonged acute rehabilitation to improve mobility and (2) VR aimed at creating employment opportunities.

### Poor balance

In this study, the participants reported challenges to maintain their balance which affected their mobility.

‘… my balance is completely out….’ (Participant B, male, unemployed)

‘Yeah, yeah, to balance myself [*is a problem*] yeah.’ (Participant F, male, unemployed)

### Standing for longer periods of time

Even though standing is a relatively simple functional task for a person with an LLA, the participants reported that they struggled with standing for a long period of time.

‘No. I couldn’t stand for a long time.’ (Participant E, male, unemployed)

‘Very difficult, because I had to hold onto something.’ (Participant B, male, unemployed)

#### Difficulty in walking long distances

Furthermore, participants who could not stand for long periods of time also had difficulty in walking long distances.

‘No, I can’t walk so long, because I get tired quickly.’ (Participant C, male, unemployed)

‘[*Walking makes me*] very tired.’ (Participant A, female, unemployed)

#### Fear of falling

Another factor that arose when participants were asked to explain why they have challenges with mobility was the fear of falling. Some participants were eager to mobilise initially, but experienced a fall, which left them too scared to try again.

‘Yes, it does help, but I’m scared that I’m going to fall.’ (Participant A, female, unemployed)

‘Because I can fall you see.’ (Participant F, female, unemployed)

### Inadequacy of acute rehabilitation

Participants in this study generally felt that their acute post-operative rehabilitation was too short to prepare them adequately for discharge to their home environment and in some instances even basic mobility. In some instances, participants were only issued with crutches on discharge from hospital and not taught how to be functionally mobile with them. Therefore, because of the early discharge the participants struggled with mobility which prevented them from participating in their daily activities. This also caused a participation barrier as they were not adequately prepared on how to face the challenges at home and in the community. These challenges included lack of ability to walk long distances, basic mobility and use of crutches. This further hindered their attempts to return to formal employment.

‘No, no, no they never taught me, but I have ramps now my uncle made a ramp.’

(Participant A, female, unemployed)

‘No I didn’t get any physio.’ (Participant G, male, unemployed)

### Vocational rehabilitation aimed at employment opportunities

Most of the participants in the study struggled with mobility. In addition, the majority of the participants who participated in interviews were retired or unemployed; hence, information on VR needs was limited. Even though 75% (*n* = 6) of the participants indicated that they wanted to work, they either struggled to find alternative employment opportunities after losing their limb or there was simply no employment available for them. Considering SA’s generally high rate of unemployment, the job market is very competitive and an additional challenge for persons with a physical disability. Participants, however, expressed that rehabilitation aimed at improving access to the job market would be greatly beneficial.

‘The only thing that I have a problem is finding a job….’ (Participant C, male, unemployed)

‘I can’t work, but I would love to. I can, If I, you see, this is a thing that lays heavy on my….’ (Participant B, male, unemployed)

## Discussion

This study aimed to address two objectives. Firstly, to determine the participation restrictions experienced by the study sample and, secondly, to explore the participation restrictions in more depth and determine the VR needs. The findings of this study provide new insights into the participation restrictions still experienced by the study sample at a minimum of 3 months post-surgery. Even though some participation restrictions are expected among persons with a unilateral LLA, the underlying reasons for these restrictions are rarely explored, but have now been voiced by the participants themselves. Even though limited information pertaining to vocational needs could be obtained, the majority of participants would have liked to work, if they had access to the opportunity. This highlights the need for VR to be an integral part of rehabilitation of persons with amputations.

In the first phase, using the WHODAS 2.0, results were similar to what is reported in other international studies; LLA significantly negatively impacted mobility in this cohort (Bragaru et al. 2011; Yazicoioglu et al. 2007). Approximately one-quarter of participants (22%) reported having a problem in participating in community activities; however, 70% reported difficulty standing and balancing, and 92% reported having difficulty in walking long distances. Walking and standing are crucial activities of daily living and play a major role in participation in society (Manini 2011). On the contrary, In contrast Yazicioglu et al. (2007) stated that participants could regain their pre-morbid functional independence with the aid of a prosthesis. Their study findings are in conflict with the results of our study because even with the assistance of the prosthesis, the participants of this study still had challenges with their mobility.

In the second phase of the study, telephonic interviews were conducted to explore the underlying reasons of participation restrictions and determine participants’ VR needs. Participants struggled with integration back into society. The participants provided feedback on the inadequate acute rehabilitation received as a major factor that hampered their re-integration into society. The inadequate acute rehabilitation also potentially hindered the employment opportunities and hobbies available to participants. Burger and Marincek (2007) reported conflicting results where two-thirds of persons with an LLA in their study sample were still able to return to work, with a slight adjustment in the environment. Returning to work after an LLA requires a good self-esteem and a series of rehabilitation and adjustments to a person’s surroundings, which participants in the current study did not receive. In this study, the main vocational need identified was employment opportunities. Based on the data collected, it was found that many participants wanted to work but could not do so because of inadequate rehabilitation or employment opportunities.

This study recommends that persons with an amputation should receive adequate rehabilitation while in hospital to adequately prepare them to re-integrate into society. Alternatively, persons with a unilateral LLA should be transferred to a subacute rehabilitation facility until they can achieve independent functional mobility in the community. Independent mobility, and a home visit to evaluate the home environment, should be considered the minimum requirements for discharge from rehabilitation. Where appropriate, persons with an LLA should be screened and provided access to prosthetics, which could increase mobility and, consequently, participation in society. Rehabilitation professionals should also emphasise the re-integration into society and include a VR assessment as part of therapy. The high unemployment rate in SA (World Bank 2017) may prove to be a significant challenge to providing employment opportunities for persons with physical disabilities, but health professionals should adequately rehabilitate persons with an LLA to have a fair chance to access the job market, as well as advocate for more opportunities.

## Study limitations

Because of the long periods of delays in different facilities, and limited permission to only access two healthcare institutions (tertiary hospital and subacute hospital), only 50 participants were recruited for the study over a period of 12 months. Limited information was also captured in the demographic survey pertaining to types of employment. Participants were only asked if they were employed or not.

In addition to the low participant sample size in the first phase of the study, many participants also refused to participate in the telephonic interviews in the second phase, which further limited the available sample for the qualitative phase of the study. Another factor that limited the available sample size for the second phase of the study was challenges because of missing or incorrect contact numbers or participants not answering their phones. Even though data saturation was reached in the qualitative phase, a study sample of 10–12 participants in the interviews would have been more representative of the initial study sample. Therefore, eight participants are not representative of the 50 surveyed, as four of the eight participants were white people and only one was a female participant. It should be noted that the results should therefore be interpreted with caution. It, however, provides a glimpse of insight into the challenges experienced with participation in society post-LLA.

## Conclusion

The difficulty in mobilising independently negatively affected the study cohort’s participation in society and vocational activities such as work. The underlying reasons reported, which could be addressed by adequate rehabilitation, were poor balance, poor cardiovascular endurance and the fear of falling that developed after the amputation. In addition, the participants reported inadequate rehabilitation and assistance in employment. The deeper insight gained into the underlying reasons for these participation restrictions can potentially assist to inform and develop patient-centred VR programmes for persons with a unilateral LLA.

## Appendix 1

### Telephonic interview questions

- Participation restrictions
  1. Can you please tell me what you are struggling with since you have lost your leg?
  2. Did you receive rehabilitation (exercises) in the hospital?
    - Did you feel that it helped you to be more independent at home?
    - Can you recommend anything else that might help other people that should have been included?
  3. Do you have a problem going out into your community?
    - Did you face any problems when you’re doing tasks in your community?
    - Are there any barriers that hinder your assistive devices or movement?
    - Do you feel ashamed because of your disability?
    - Do they look down on you because of your loss of limb?
    - How did your family respond to your disability?
    - What do you want to do when you want to relax?
      - Do you have any hobbies or fun activities?
      - Is there anything that makes it difficult to enjoy that activity?
- Self-care at home:
  1. Do you have challenges when staying at home alone?
    - What makes it difficult for you to stay alone?
    - What do you struggle with when you stay alone?
    - Do you feel safe to do normal activities when no one is around you?
      - For example, bath/make tea/cook
    - Does your house require a lot of work for you to do?
    - Are you able to freely move around in your house?
      - With/without your assistive device
- Life activities:
  1. Is there any chores/house work that you cannot do after your amputation?
  2. *Link the below*
    - What do you think you need to overcome the struggles?
    - Are there any struggles with the assistive device that you have currently?
    - What type of household responsibilities do you struggle with?
    - What hinders you is in doing the household tasks?
    - What is preventing you in doing your task quickly?
    - Does that affect your work/go to school:
      - Is there any difficulties in attending school/work?
      - What would you recommend therapists do for improving your working abilities?
- Getting around:
  1. Do you have any challenges when you travel to different places?
    - Public transport
    - How far
    - What makes it difficult to stand for a long period of time?
  2. Is it challenging stand up from sitting?
    - Why do you struggle/what makes it difficult to stand up from sitting?
  3. Is it challenging to stand up?
    - How long can you generally stand for before you need to sit down?
    - What do you struggle with when you try to move around at home?
    - What makes it difficult for you to walk long distances?
      - Why do you need this break?
      - Why do you struggle
